# Eradication of Isolated Para-Aortic Nodal Recurrence in a Patient with an Advanced High Grade Serous Ovarian Carcinoma: Our Experience and Review of Literature

**DOI:** 10.3390/medicina58020244

**Published:** 2022-02-06

**Authors:** Raffaele Tinelli, Miriam Dellino, Luigi Nappi, Felice Sorrentino, Maurizio Nicola D’Alterio, Stefano Angioni, Giorgio Bogani, Salvatore Pisconti, Erica Silvestris

**Affiliations:** 1 Department of Obstetrics and Gynecology, “Valle d’Itria” Hospital, 74015 Martina Franca, Italy; 2 Department of Gynecology Oncology, IRCSS Giovanni Paolo II, 70124 Bari, Italy; miriamdellino@hotmail.it (M.D.); ericasilvestris85@gmail.com (E.S.); 3 Department of Medical and Surgical Sciences, Institute of Obstetrics and Gynaecology, University of Foggia, 71121 Foggia, Italy; luigi.nappi@unifg.it (L.N.); felice.sorrentino.1983@gmail.com (F.S.); 4 Department of Surgical Science, University of Cagliari, Cittadella Universitaria Blocco I, Asse Didattico Medicina P2, Monserrato, 09042 Cagliari, Italy; mauridalte84@gmail.com (M.N.D.); sangioni@yahoo.it (S.A.); 5 Department of Obstetrics and Gynecology, University Medical School La Sapienza, 00185 Rome, Italy; bogani.giorgio@gmail.com; 6 Department of Medical Oncology, National Oncology Institute Moscati, 74010 Taranto, Italy; salvatore.pisconti@asl.taranto.it

**Keywords:** aortic, lymphadenectomy, lymphnodes, ovarian cancer, recurrence, recurrent ovarian cancer, relapse, secondary cytoreduction

## Abstract

We report a case report regarding the eradication of isolated lymph-nodal para-aortic recurrence in the aortic region down the left renal vein (LRV) in a patient treated two years earlier in another hospital for a FIGO stage IC2 high-grade serous ovarian carcinoma with a video showing the para-aortic space after eradication of the metastatic tissue. A 66 year-old woman was admitted 24 months after the initial surgical procedure for an increased Ca 125 level and CT scan that revealed a 3 cm para-aortic infrarenal lymph-nodal recurrence that was confirmed by PET/CT scan. A secondary cytoreductive surgery (SCS) with a para-aortic lymph-nodal dissection of the tissue down the LRV and radical omentectomy were performed: during the cytoreduction, the right hemicolon was mobilized. The anterior surface of the inferior vena cava (IVC), aorta and LRV were exposed. The metastatic lymph nodes were detected in the para-ortic space down the proximal part of the LRV and eradicated; an en bloc infrarenal lymph-node dissection from the aortocaval region was performed. The operative time during the surgical procedure was 212 min with a blood loss of 120 mL. No intra- and postoperative complications, including ureteral or vascular injury or renal dysfunction, occurred. At histological examination, three dissected lymph nodes were positive for metastasis, and the patient was discharged five days after laparotomy without side effects and underwent chemotherapy 3 weeks later; after a follow-up of 42 months, no recurrence was detected. In conclusion, secondary debulking surgery can be considered a safe and effective therapeutic option for the management of recurrences, although long-term follow-ups are necessary to evaluate the overall oncologic outcomes of this procedure.

## 1. Introduction

A secondary debulking surgery with resection of a suspicious recurrence should be considered in women with a high-grade serous ovarian carcinoma to complete cytoreduction [1,2,3]; secondary cytoreductive surgery (SCS) for recurrent ovarian cancer presenting as isolated lymph node metastases is associated with an increased long-term survival if complete SCS is performed [1], although the removal of aortic metastatic lymph nodes could be associated with severe bleeding if performed by surgeons not trained in extensive oncological procedures. 

Disease relapse is the primary cause of death from ovarian carcinoma. Isolated lymph node relapse (ILNR) is a rare pattern of ovarian carcinoma recurrence, with a reported median post-recurrence survival of 2.5 to four years [4]. 

In this article, we reported a case regarding the eradication of isolated lymph-nodal para-aortic recurrence with a video showing the para-aortic space after eradication of the metastatic tissue without any postoperative complications in a patient treated two years earlier in another hospital for a high-grade serous ovarian carcinoma.

## 2. Case Report

A 66 year-old postmenopausal patient, with a history of debulking surgery for a high-grade serous ovarian carcinoma two years earlier in a secondary hospital, was admitted at our department for a suspected isolated lymph-nodal para-aortic recurrence.

The patient was managed for FIGO stage IC2 two years earlier with a type A radical hysterectomy (according to the Querleu–Morrow classification) [5], bilateral salpingo-oophorectomy, omentectomy, pelvic lymphadenectomy and an adjuvant chemotherapy with six cycles of paclitaxel and carboplatin. 

24 months after the initial surgical procedure, an increased Ca 125 level and CT scan performed at follow-up revealed a 3 cm para-aortic infrarenal lymph-nodal recurrence that was confirmed by PET/CT scan.

**Figure 1:**
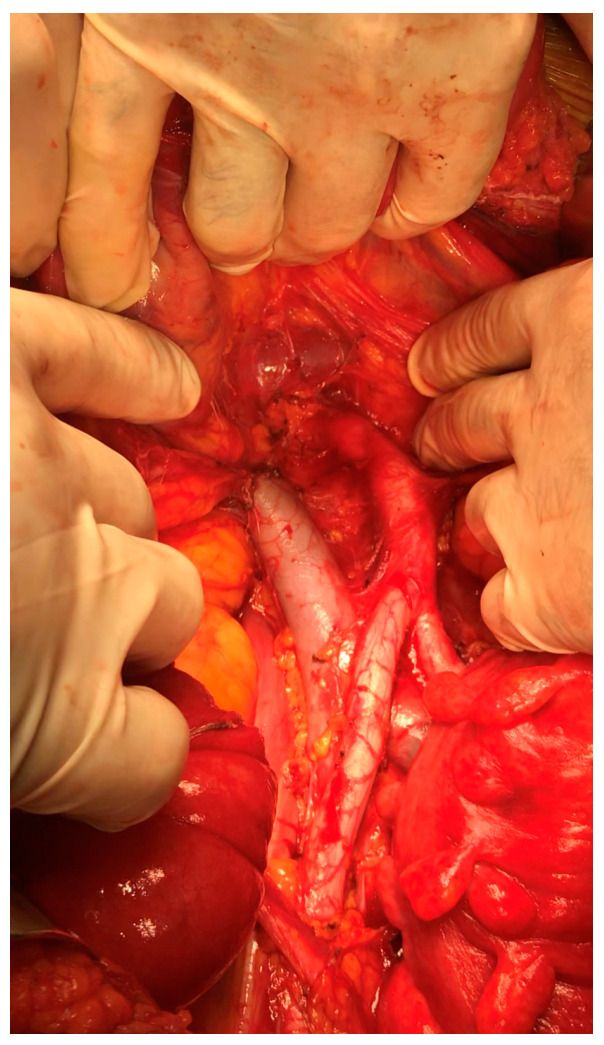
Para-aortic space after eradication of the nodal recurrence.

A secondary debulking open surgery with a para-aortic lymph-nodal radical dissection of the tissue down the LRV and a radical omentectomy were performed: during the cytoreduction, the right hemicolon was separated from the retroperitoneum and was mobilized. The anterior surface of the inferior vena cava (IVC), aorta and LRV were exposed. The metastatic lymph nodes were detected in the para-ortic space down the proximal part of the LRV and eradicated; an en bloc resection of the infrarenal lymph nodes in the aortocaval region was performed, as shown in Figure 1. The operative time during the surgical procedure was 212 min, and the estimated blood loss was 120 mL. 

No intra- or postoperative complications, including ureteral or vascular injury or renal dysfunction, occurred. At histological examination, three dissected lymph nodes were positive for metastasis, and the patient was regularly discharged five days after the laparotomic procedure without any significative side effects and underwent chemotherapy three weeks later; actually, after a follow-up of 42 months no recurrence was detected.

## 3. Discussion

Chemotherapy has always been considered the treatment of choice for recurrent epithelial ovarian cancer (EOC), but its use in isolated lymph-nodal relapse (ILNR) is still debated. Secondary cytoreductive surgery (SCS) involves high risks of vascular injury due to the proximity of major intra-abdominal and pelvic blood vessels. 

Vascular repairs during gynecologic oncologic surgery are often performed for iatrogenic injuries or during extensive oncologic resections and are increasing in frequency [4,5,6,7].

Pelvic and aortic lymphadenectomy are performed during surgical treatment of gynecological malignancies for their prognostic and therapeutic significance [1]. These procedures are performed close to multiple vascular structures with a high risk of intraoperative hemorrhage. Vascular injuries are the most potentially catastrophic complications, frequently involving iliac veins [8,9] due to their complicated distributions, anatomical variability and their positions posterior to the common iliac artery bifurcation. 

Several retrospective studies confirmed the advantages of SCS in patients with a long disease-free interval, resectable disease (based on imaging), an absence of ascites, a limited number of metastatic sites and a good performance status [10]. However, the frequency of isolated lymph node recurrence (ILNR) is actually rare (between 1% and 6%). In these patients, SCS could be of particular benefit [11]. 

Blanchard et al. reported their experience with 27 patients with an ILNR among 640 patients. They observed that after initial surgical treatment, the median progression-free survival (PFS) was 26 months. There was no difference in the two-year survival after ILNR between the groups with early relapse (before 24 months) and late relapse (after 24 months). The time to relapse may not have its usual prognostic value and may not have the same value as in other sites of relapse. The prognosis of ILNR seems better than the prognosis of metastatic recurrence of EOC at other sites. Immediate or delayed therapy should be discussed in cases of asymptomatic ILNR [12].

Santillan et al analyzed the outcomes of SCS for the isolated lymph-nodal relapse of EOC and the subsequent survival rate. Twenty-five patients with epithelial ovarian cancer who underwent SCS for isolated lymph node relapse were identified from tumor registry databases. All patients received platinum-based chemotherapy following primary surgery. With SCS, a residual disease ≤1 cm was achieved in 100% of patients. At a median post-recurrence follow-up time of 19 months, eight patients (32%) have died of the disease, seven (28%) are alive with disease, and 10 (40%) patients are alive without evidence of disease. The median post-recurrence OS after SCS for recurrent nodal disease was 37 months. They concluded that an optimal SCS for recurrent epithelial ovarian cancer presenting as isolated node metastases is feasible in the majority of cases with a favorable long-term survival outcome [13].

In their retrospective study, Legge et al. analyzed 301 patients with recurrence after optimal cytoreductive surgery for epithelial ovarian cancer, of whom 32 had isolated lymph node recurrence and were identified from tumor registry databases. They observed that, although these results need to be confirmed with long-term multicenter studies, ILNR seems to represent a less aggressive pattern of disease relapse; however, peritoneal spreading after the first ILNR documentation is frequently associated with a poor outcome [14].

The DESKTOP trial, based on data from a retrospective analysis of hospital records on 267 patients, created a panel of criteria for selecting patients who might benefit from surgery in recurrent ovarian cancer and a score model for complete resectability based on performance status, absence of ascites, and outcome of primary surgery/initial FIGO stage [1]. A complete resection was associated with significantly longer survival compared with surgery leaving any postoperative residuals [median 45.2 vs. 19.7 months]. Variables associated with a complete resection were a good performance status (PS), the (FIGO) stage at initial diagnosis (FIGO I/II vs. III/IV, *p* = 0.036), no residual tumor after the first surgery and the absence of ascites; these could predict a complete resection in 79% of patients. They concluded that only a complete resection was associated with prolonged survival in recurrent ovarian cancer (1). In the DESKTOP II prospective trial, all consecutive patients with platinum-sensitive first or second relapse were enrolled. The score was applied to all patients, and a total of 129 patients with a positive score and first relapse were operated on. The rate of complete resection was 76%, thus confirming the validity of this score regarding the positive prediction of complete resectability. They concluded that this score was the first validated instrument for predicting the surgical outcome in recurrent ovarian cancer [2].

In their multicenter study, Gadducci et al. reported 69 patients with epithelial ovarian cancer who were clinically or pathologically free of disease after primary therapy and who developed an isolated lymph node recurrence. 

They observed that patients who underwent surgery plus chemotherapy had a 72% reduction in the risk of death after recurrence and a 75% reduction in the risk of death after initial diagnosis when compared with those treated with chemotherapy alone. They concluded that SCS increased survival in epithelial ovarian cancer patients with an apparently isolated lymph node recurrence [10].

Another multicenter study by Ferrero et al. that included 73 patients with ROC evaluated the outcomes of SCS in patients with lymph-nodal relapse (LR). They concluded that secondary surgery for isolated LR of ovarian cancer was safe, effective and associated with a low morbidity and an improved outcome [11,12,13,14,15].

A recent review by Marchetti et al. compared SCS followed by systemic therapy with systemic therapy alone for the management of patients with recurrent ovarian cancer. SCS was associated with a significantly better progression-free survival (PFS) than chemotherapy alone for the complete resection group [16]. 

Szczesny et al. enrolled 397 patients who had a primary diagnosis of FIGO stage I–IV EOC and received primary surgery with no residuals followed by platinum-based chemotherapy; they had the first recurrence six or more months after the primary platinum-based chemotherapy and received the secondary treatment with either SCS and chemotherapy (SCS plus platinum-based chemotherapy group) or platinum-based chemotherapy alone (platinum-based chemotherapy group). There were 75 patients in the SCS plus platinum-based chemotherapy group, in whom a complete resection was achieved for 60 (80%), and there were 322 patients in the platinum-based chemotherapy group. Improvements of PFS and OS were observed in the SCS plus platinum-based chemotherapy group when compared with the platinum-based chemotherapy group. The authors observed a survival benefit in patients with no residuals at SCS and concluded that in selected patients with no residuals after primary surgery and a recurrent, platinum-sensitive tumor, a complete resection of recurrence at SCS improved progression-free survival and overall survival [17,18,19,20,21,22]. 

However, Bickell et al. analyzed 2038 women who underwent a primary debulking surgery with six cycles of chemotherapy and experienced a remission. 1635 of 2038 (80%) women received a treatment for recurrence, 72% of whom were treated with chemotherapy only, 16% with surgery and chemotherapy, while 12% received hospice care. The authors concluded that survival among women with recurrence was greater for those treated with surgery and chemotherapy compared with chemotherapy alone; if these data are confirmed by pending randomized trial results, SCS could be considered a standard of care [21].

In a series of 118 patients with recurrent ovarian cancer (ROC), So et al. evaluated the following two treatment options: secondary cytoreductive surgery (SCS) combined with chemotherapy and chemotherapy alone. They confirmed that SCS combined with chemotherapy offered better PFS and OS than chemotherapy alone in first platinum-sensitive ROC patients [22,23]. 

Hollis et al. performed a molecular characterization of 98 patients with recurrent ovarian cancer who subsequently experienced isolated relapse: 49 isolated lymph node relapse ovarian carcinoma patients were identified and matched to 49 extranodal relapses. The authors concluded that isolated lymph node relapse ovarian carcinoma represented a distinct clinical entity with a favorable outcome and with significantly prolonged postrecurrence survival and overall survival when compared to extranodal relapse. Diagnostic tumor material from isolated lymph node recurrence demonstrated a greater CD3+ and CD8+ cell infiltration, which may have contributed to the more indolent disease course of isolated lymph node relapse, while there was no depletion of BRCA1/2 mutation in the isolated lymph node relapse [4,24]. 

Finally, data from the final analysis of DESKTOP III presented at ASCO this year showed that ROC patients with a positive AGOscore [performance status Eastern Cooperative Oncology Group (ECOG) = 0, ascites ≤ 500 cc, and no residual tumor at initial surgery] who received SCS and CHT resulted in showing a significantly longer PFS compared with those who received CHT alone (the median PFS was 18.4 vs. 14 months, respectively; <0.001). The primary endpoint analysis showed a median overall survival (OS) of 53.7 months with and 46.0 months without surgery (*p* value = 0.02), with an even more significant difference in patients with a complete tumor cytoreduction (CTC) compared to patients without surgery (the median OS was 61.9 vs. 46.0 months) [18]. 

## 4. Conclusions 

Our experience confirms that SCS can be considered a safe and effective therapeutic option for the management of isolated lymph-nodal recurrence of ovarian cancers, with a significantly low morbidity and postoperative hospitalization, although it should be reserved for oncologic surgeons trained in extensive surgical procedures; furthermore, large multicenter randomized clinical trials with longer follow-ups are necessary to confirm the overall oncologic outcomes of this procedure.

## Data Availability

Not applicable.

## Availability of Data and Materials

All data generated or analyzed during this study are included in this published article.
